# Brief Report: Characteristics and Needs of Persons Admitted to an Inpatient Psychiatric Hospital With Workers' Compensation Coverage

**DOI:** 10.3389/fpsyt.2021.673123

**Published:** 2021-05-28

**Authors:** Mary Grace Herring, Lynn Martin, Vicki L. Kristman

**Affiliations:** ^1^Department of Health Sciences, Lakehead University, Thunder Bay, ON, Canada; ^2^Enhancing Prevention of Injury & Disability (EPID)@Work Research Institute, Lakehead University, Thunder Bay, ON, Canada

**Keywords:** interRAI, mental health, inpatient psychiatry, worker, trauma, pain, substance use, depression

## Abstract

The rise of mental health issues in the workplace is widely known. Though mental health issues were not covered by the Workplace Safety Insurance Board (WSIB) in Ontario (Canada) until 2018, it was listed as responsible for payment of inpatient psychiatric hospital stays between 2006 and 2016. This population-level observational analytic study compares the clinical and service needs of 1,091 individuals admitted to inpatient psychiatry with WSIB coverage to all other admissions (*n* = 449,128). Secondary analysis was based on the interRAI Mental Health assessment. The WSIB group differed from all other admissions on almost all characteristics considered. Most notably, depression (65.08 vs. 57.02%), traumatic life events (25.48 vs. 15.58%), substance use (58.02 vs. 46.92%), daily pain (38.31 vs. 12.15%) and sleep disturbance (48.95 vs. 37.12%) were much higher in the WSIB group. Females with WSIB coverage had more depression (74.36 vs. 59.91%) and traumatic life events (30.00 vs. 22.97%), whereas males had more substance issues (63.62 vs. 47.95%). In addition, persons under the age of 55 had more substance issues (<25 = 75.47%; 25–54 = 61.64%: 55 ± 40.54%) and traumatic life events (<25 = 26.41%; 25–54 = 28.18%; 55 ± 15.31%), while those 25–54 years had more daily pain (41.67% vs. <25 = 3.77% and 55 ± 34.23%) and sleep disturbance (50.74% vs. <25 = 33.96% and 55 ± 45.94%). All variables differed significantly by sex and age within the comparison group, though not always following the patterns observed in the WSIB group. Future research examining mental health needs and outcomes among injured workers receiving inpatient psychiatric services is needed, and should take into account sex and age.

## Introduction

That mental health conditions are on the rise is widely known. These affect hundreds of millions of people internationally, and are now recognized among the leading causes of disability (1). By 2030, the global cost of mental illness will surpass six trillion dollars (2). In Canada, the annual economic burden of mental illness is ~50 billion dollars, and is projected to reach 307 billion by 2041 (2).

The rise of mental health issues in the workplace is equally widely known. A systematic review and meta-analysis found that ~18% of workers met the criteria for common mental health conditions (i.e., mood, anxiety, substance-related disorders), with a lifetime prevalence of just under 30% (3). It is not surprising then, that programs and policies targeting mental health in the workplace have become a focus for employers and governments around the world.

In Canada, workers' compensation started with the 1913 Meredith Report; it outlined an arrangement in which workers ceded their right to sue in return for benefits (4). The main tenets of the original workers' compensation laws still exist: no-fault compensation, collective liability, security of payment, exclusive jurisdiction, and independent board. In spite of having common principles, Canadian provinces and territories have their own Workers' Compensation Board (WCB), apart from the Northwest Territories and Nunavut, which share a WCB. Governments in each jurisdiction create Workers Compensation legislation, which is administered by the WCB. The benefits provided to workers most commonly fall into one of five categories: health care, wage loss benefits, permanent disability benefits, fatal and dependency benefits (survivor benefits), and rehabilitation. The levels of coverage and benefit amounts vary by WCB. For example, the percentage of earnings that benefits are based on vary: 85% net in Prince Edward Island and Ontario, and 90% net in Quebec, Manitoba, Saskatchewan, Alberta, British Columbia, and Northwest Territories and Nunavut. Federally, all government employees are governed under the Federal Government Employees Compensation Act.

While for some, mental health conditions may not originate in the workplace, there is considerable evidence that the workplace may itself contribute to mental illness (5). This led to changes in Ontario's Workplace Safety and Insurance Board (WSIB); its new chronic mental stress policy and revised traumatic mental stress policy came into effect on January 1, 2018. Ontario's WSIB previously compensated chronic mental health injuries until Bill 99 removed this coverage in 1998. In 2016, Bill 163 was passed that recognized post-traumatic stress disorder in first responders to be a work-related injury, unless proven otherwise. However, there was still no coverage for mental health injuries developed over time. In 2017, the Ontario government passed Bill 177, which amended the Act to cover chronic and traumatic mental stress as long as the stress is not caused by “decisions or actions of the worker's employer relating to the worker's employment, including a decision to change the work to be performed or the working conditions, to discipline the worker or to terminate the employment” (6).

Though mental health issues were not explicitly covered by the WSIB until 2018, it was listed as responsible for payment of inpatient psychiatric stays between January 1, 2006 and December 31, 2016. This study compares the clinical and service needs of the 1,091 individuals admitted to inpatient psychiatry in this 11-year period to all other admissions. Given that the nature and experience of work-related injuries and disabilities are known to differ by sex and age (7, 8), within and between-group comparisons by age and sex will be made.

## Materials and Methods

### Data

This observational analytic study employed secondary analysis of anonymized population-level clinical data in the Ontario Mental Health Reporting System (OMHRS) collected between January 1, 2006 and December 31, 2016 on all persons admitted to an adult inpatient psychiatric bed or unit. Anonymized data are held on a secure server at the University of Waterloo as part of a data-sharing agreement between the Ontario Ministry of Health and interRAI, a not-for-profit research consortium. Exemption from review for secondary analysis of anonymized data was granted by the Lakehead University Research Ethics Board, as per the Tri-Council Policy Statement (9).

### Instrument

Data are based on the RAI Mental Health assessment (10–12), which contains over 300 items targeting key domains: personal information, mental health service history, mental state, substance use, injurious behaviors, social roles and relationships, psychiatric diagnoses, cognition, functioning, health conditions and medical diagnoses, medications, life stressors, service use, and informal supports. Items are also grouped into various scales, algorithms, and protocols framed on recovery principles that support evidence-informed individual-level care planning and shared decision-making processes about services (12, 13). It is completed at admission and discharge (if the stay lasts at least 7 days); reassessments are completed every 90 days if still in hospital.

### Study Population/Variables

Admission assessments were completed on 449,128 unique individuals in the study period; if individuals had more than one admission, the most recent assessment was used. The group of interest are workers who experience mental health issues, defined as those for whom WCB/WSIB was listed as a source responsible for payment. The comparison group represents all other admissions.

Items related to personal information (i.e., age, sex, marital status, living arrangements) and stay (i.e., reasons for admission, length of stay) were used, as were DSM-V diagnostic categories (actual or provisional diagnoses). All embedded scales available in the assessment were used to describe clinical characteristics: Activities of Daily Living Hierarchy (ADLH) (14), Aggressive Behavior Scale (ABS) (15), Cognitive Performance Scale (CPS) (16), Depression Rating Scale (DRS) (17), substance use (CAGE scale) (12), Mania Scale 10, Positive Symptoms Scale (12), Social Withdrawal Scale (SWS, formerly called Negative Symptoms Scale, NSS) (12), and Pain Scale (18). Previously-established cut-offs were used for each scale to indicate problems in that area (e.g., higher severity, frequency). A succinct description of the items and coding used in embedded scales is available elsewhere (19).

Just as scales represent the combination of items to provide information on overall status in different areas, items are also combined to flag issues. These are called clinical assessment protocols (CAPs), and identify needs related to safety (i.e., harm to self and others, suicidality and purposeful self-harm, self-care), social life (i.e., social relationships, informal support, support systems for discharge, interpersonal conflict, traumatic life events, criminal activity), economic issues (i.e., personal finance, education, and employment), autonomy (i.e., control interventions, medication management and adherence, rehospitalization), and health promotion (i.e., smoking, substance use, weight management, exercise, sleep disturbance, pain, falls) (12, 13, 20).

### Analysis

The prevalence of persons admitted with WSIB coverage is shown annually and overall. Descriptive statistics (%, mean, standard deviation) inform on all variables, and relevant tests of significance were used to report on differences within and between groups (chi-square, *t*-test, ANOVA). Given the very large number of admission assessments, a strict Type I error rate was used (i.e., alpha = 0.001); only findings that meet this criteria are described. The same reporting format is used for all results: (a) description of between group differences, (b) description of within-WSIB group differences by age and sex, and (c) description of within-Other group differences by age and sex that differ from what was found in the WSIB group. As such, within-Other group differences by sex and age that are not described are similar to those within the WSIB group.

## Results

There were 1,091 assessments that had WSIB listed among responsible sources of payment, representing 0.24% of all admissions (Table 1). Such admissions ranged between 56 and 129 per year; they decreased between 2006 and 2010 and then steadily increased.

**Table 1:** Number of WSIB admissions between January 1, 2006 and December 31, 2016.

**Year**	**Total admissions (*N*)**	**WCB/WSIB N (% of all admissions)**
2006	38,655	103 (0.27%)
2007	38,984	88 (0.23%)
2008	38,399	90 (0.23%)
2009	39,426	83 (0.21%)
2010	40,206	56 (0.14%)
2011	40,636	95 (0.23%)
2012	41,551	104 (0.25%)
2013	41,490	107 (0.26%)
2014	42,432	109 (0.26%)
2015	43,110	127 (0.29%)
2016	44,239	129 (0.29%)
Total	449,128	1,091 (0.24%)

### Study Population Characteristics

Table 2 shows personal and admission characteristics; multiple reasons may be listed for each admission.

**Table 2:** Univariate distribution (%) of personal characteristics of persons admitted overall, and by WSIB status, sex, and age.

	**WSIB**						**Other**					
	**All**	**Sex**		**Age group**			**All**	Sex^*^		**Age group**		
		**F**	**M**	** <25**	**25-54**	**55+**		**F**	**M**	** <25**	**25-54**	**55+**
***N***	1,091	401	734	53	816	222	448,037	219,504	228,408	73,723	265,205	109,109
**Marital status**^**a,c,d,e**^												
Never married	34.83	35.65	34.38	94.34	36.15	15.77	54.16	44.71	63.22	95.83	55.34	23.13
Married	35.84	34.36	36.66	1.89	36.40	41.89	22.71	26.82	18.77	1.83	22.50	37.33
Partner	4.86	4.87	4.85	1.89	5.51	3.15	3.43	4.04	2.85	1.57	4.36	2.44
Widowed	2.38	3.59	1.71	1.89	0.49	9.46	4.10	6.29	2.00	0.07	1.04	14.27
Separated/Divorced	22.09	21.54	22.39	0	21.45	29.73	15.60	18.14	13.16	0.72	16.76	22.83
**Who lived with**^**a,b,c,d,e**^												
Alone	31.62	32.31	31.24	15.09	32.11	33.78	32.41	31.96	32.84	16.78	34.64	37.54
Spouse only	16.50	17.18	16.12	1.89	14.46	27.48	11.96	14.54	9.48	2.11	9.55	24.49
Spouse + other/s	22.18	17.18	24.96	5.66	24.75	16.67	11.17	13.52	8.90	2.12	15.12	7.68
Child (not spouse)	5.32	9.49	3.00	0	5.39	6.31	4.55	7.88	1.35	1.04	5.08	5.63
Other relative	20.26	20.77	19.97	69.81	19.98	9.46	28.89	23.48	34.10	69.07	26.25	8.17
Non-relative(s)	4.12	3.08	4.71	7.55	3.31	6.31	11.02	8.62	13.33	8.89	9.36	16.49
**Admitted from**^**a,c,d,e**^												
Home/apt/room	76.71	79.93	75.00	64.71	78.69	72.41	59.22	61.90	56.67	58.59	61.17	55.13
Psychiatric hospital/unit	1.27	1.46	1.16	2.94	1.36	0.057	2.67	2.44	2.89	2.65	2.91	2.41
Homeless	1.39	0.36	1.94	2.94	1.20	1.72	3.25	2.27	4.19	3.40	3.92	1.59
Acute unit/hospital	12.66	11.31	13.37	26.47	11.51	13.79	23.33	23.67	23.00	22.00	23.93	26.91
Other	7.97	6.94	8.53	0	4.30	5.17	11.53	9.72	9.38	3.88	3.83	3.74
Reasons for admission^**^												
Threat/danger to self^a,b,d,e^	44.18	49.47	41.08	58.49	42.77	49.95	48.36	50.24	46.54	57.77	48.55	41.52
Threat/danger to others^a,c,d,e^	13.66	11.03	15.12	32.08	12.01	15.32	20.17	13.69	26.40	24.95	19.42	18.76
Inability to care for self^a,b,c,d,e^	23.92	31.03	19.97	28.30	21.69	31.08	40.26	41.01	39.54	37.87	49.35	35.38
Addiction/dependency^a,b,c,d,e^	38.86	29.23	44.22	41.51	42.16	26.13	25.27	18.94	31.35	29.91	28.73	3.72
Psychiatric symptoms^a,b,c,d,e^	69.94	73.59	67.90	62.26	71.94	64.41	73.14	75.79	70.59	72.11	73.22	73.64
Justice/forensic system^a,b,d,e^	3.67	1.79	4.71	3.77	3.92	2.70	6.36	3.00	9.59	7.64	7.41	2.93

* *An additional N = 125 identified their sex as “other”*;

** *Multiple reasons may be listed for each admission*;

a *Significant difference between the WSIB and Other group (p < 0.001)*;

b *Significant difference by sex within the WSIB group (p < 0.001)*;

c *Significant difference by age category within the WSIB group (p < 0.001)*;

d *Significant difference by sex within the Other group (p < 0.001)*;

e *Significant difference by age category within the Other group (p < 0.001)*.

#### Between Group Differences

The WSIB mean age was higher [45.37 years (SD = 12.4) vs. 42.91 years (SD = 17.0); *p* > 0.001] than the other group. The majority of people in both groups were male (WSIB: 64.25%; *p* < 0.001; Other: 50.99%; *p* < 0.001), equal proportions lived alone and the majority lived in a private home. However, more people in the WSIB group were married, lived with a spouse and other(s), whereas more in the other group had never been married, lived with non-relatives or other relatives, and had been admitted from an acute or other setting. The WSIB group was less often admitted for all reasons listed, with one exception: this group had more admissions due to problems with addiction or dependency. The mean length of stay was about 4 days, and did not significantly differ between groups [WSIB: 3.89 days (SD = 10.9); Other: 4.05 days (SD = 18.9); *p* = 0.77].

#### Within-WSIB Group Differences by Sex and Age

Within the WSIB group, the youngest group (i.e., under 25 years) tended to have never been married, whereas most in the oldest group (i.e., 55 years or more) were married; there were equal proportions among those 25-54 years who had never been married and who were currently married. Females more often lived with a child only compared to males, the youngest with other relatives (e.g., parent or guardian), and the two older groups most often lived alone. Females were more likely to be admitted due to threat or danger to self, inability to care for self, and specific psychiatric symptoms. Males were more often admitted due to problems with addiction or dependency and involvement with the criminal justice system. The youngest group was most often admitted for being a danger to others, while admission due to inability to care for self was highest among the oldest group. Admission for specific psychiatric symptoms was most frequent among those aged 25 to 54 years; the proportion admitted for addiction and dependency was similar among those under 25 years and between 25 and 54 years. There were no sex differences for mean age (*p* = 0.09), marital status (*p* = 0.16), place admitted from (*p* = 0.22), admission for threat/danger to others (*p* = 0.06), and mean length of stay (*p* = 0.58). There were no age differences for admission due to threat or danger to self (*p* = 0.07), justice system/forensic (*p* = 0.69), other reasons (*p* = 0.26), and mean length of stay (*p* = 0.22).

#### Within-Other Group Differences by Sex and Age

Some notable differences existed by sex and age in the other group. Compared to males, females were older, and more commonly lived with their spouse and children. They were also more often admitted from a private home, and fewer had been homeless. Males were more often admitted for being a danger or threat to others. Unlike the WSIB group, there were age-related differences for all reasons for admission. In particular, the youngest group was most often admitted for threat or danger to self and involvement with the justice or forensic system, while those 25-54 years were more often admitted due to inability to care for self. For their part, the oldest group was most often admitted due to specific psychiatric symptoms and for reasons other than those listed. The mean length of stay was statistically significantly higher among males and in the oldest age group, though the actual differences were minimal [4.16 days (SD = 22.9) vs. 3.95 days (SD = 13.6) among females; 4.33 days (SD = 24.5) vs 3.97 days (SD = 17.2) among 25–54 years and 4.00 days (SD = 22.9) among under 25 years].

### Clinical Characteristics

Table 3 reports on DSM-V diagnostic categories and clinical characteristics as measured by embedded scale scores.

**Table 3:** Univariate distribution (%) for clinical characteristics overall, and by WSIB status, sex, and age.

	**WSIB**						**Other**					
	**All**	**Sex**		**Age category**			**All**	Sex*		**Age category**		
		**F**	**M**	** <25**	**25-54**	**55+**		**F**	**M**	** <25**	**25-54**	**55+**
***N***	1,091	401	734	53	816	222	448,037	219,504	228,408	73,723	265,205	109,109
**DSM-IV categories**												
Substance-related^a,b,c,d,e^	36.66	25.90	42.65	16.98	40.93	25.68	24.90	17.73	31.79	29.59	28.38	13.29
Schizophrenia/psychotic^a,c,d,e^	16.41	15.13	17.12	35.85	15.32	15.77	36.27	29.80	42.48	39.30	38.66	28.38
Mood^a,b,c,d,e^	60.49	71.28	54.49	43.40	62.62	56.76	50.81	59.10	42.85	45.78	50.18	55.77
Anxiety^a,c,d,e^	31.07	31.79	30.67	24.53	34.93	18.47	12.87	15.54	10.29	12.85	13.30	11.83
Personality^d,e^	8.07	10.00	6.99	11.32	8.58	5.41	10.13	12.76	7.60	13.29	11.10	5.63
Other^**^												
Moderate or worse cognitive impairment (CPS 3+)^a,c,d,e^	4.12	3.85	4.28	7.55	2.70	8.56	8.27	8.09	8.45	5.04	5.00	18.42
Moderate or worse ADL impairment ADLH 3+)^3,4,5^	4.22	4.36	4.14	1.89	3.09	9.01	5.38	5.53	5.25	2.18	2.64	14.22
Severe aggression (ABS 5+)^a,c,d,e^	4.49	5.90	3.71	9.43	3.55	6.76	9.48	8.90	10.05	10.74	8.35	11.40
Possible depression (DRS 3+)^1,2,4,5^	65.08	74.36	59.91	54.72	66.91	60.81	57.02	65.01	49.34	54.86	56.65	59.38
Possible substance problem (CAGE 2+)^a,b,c,d,e^	25.30	15.90	30.53	28.30	27.82	15.32	17.79	13.66	21.76	19.55	20.64	9.69
Any sign of mania (Mania Scale 1+)^d,e^	54.72	57.69	53.07	62.26	54.66	53.15	56.76	56.54	56.98	59.76	56.61	55.10
Any positive symptom (PSS 1+)^a,c,d,e^	28.23	29.49	27.53	54.72	25.61	31.53	48.61	45.46	51.65	50.77	48.60	47.20
Any social withdrawal (SWS 1+)^b,d,e^	61.69	66.15	59.20	62.26	62.62	58.11	59.22	61.49	57.04	57.83	58.89	60.97
Daily pain (Pain Scale 2+)^a,c,d,e^	38.31	36.41	39.37	3.77	41.67	34.23	12.15	13.89	10.47	4.78	12.48	16.31

* *An additional N = 125 identified their sex as “other”*;

** *Includes all other possible DSM-V categories*;

a *Significant difference between the WSIB and Other group (p < 0.001)*;

b *Significant difference by sex within the WSIB group (p < 0.001)*;

c *Significant difference by age category within the WSIB group (p < 0.001)*;

d *Significant difference by sex within the Other group (p < 0.001)*;

e *Significant difference by age category within the Other group (p < 0.001)*.

#### Between Group Differences

Differences existed between the two groups for all but personality disorder diagnosis (*p* = 0.02), mania (*p* = 0.17), social withdrawal (*p* = 0.10), and ADL impairment (*p* = 0.09). A higher proportion of those in the WSIB group had substance-related, mood, and anxiety disorders, whereas schizophrenia/psychotic disorders were more prevalent in the other group. More persons in the WSIB group exceeded the cut-off scores for depression, possible substance problem, and pain; moderate or worse cognitive impairment, severe aggression, and positive symptoms were more common in the other group.

#### Within-WSIB Group Differences by Sex and Age

In the WSIB group, more females had a mood disorder diagnosis and exceeded the cut-offs for depression and social withdrawal, whereas more males had a substance-related diagnosis and exceeded the cut-off for possible substance problem. There were no sex differences for the other diagnostic categories (schizophrenia/psychotic: *p* = 0.39; anxiety: *p* = 0.70; personality: *p* = 0.08) and scales (CPS: *p* = 0.73; ADLH: *p* = 0.86; ABS: *p* = 0.09; Mania: *p* = 0.14; PSS: *p* = 0.49; Pain: *p* = 0.33). More persons in the youngest group had schizophrenia/psychotic disorder, severe aggression, possible substance problem, and positive symptoms, while more in the 25-54 years group had diagnoses related to substances, mood, and anxiety disorders and exhibited signs of daily pain. The oldest most often had moderate or worse cognitive and ADL impairment. Age differences did not exist for personality disorder diagnosis (*p* = 0.21), depression (*p* = 0.06), social withdrawal (*p* = 0.47), and mania (*p* = 0.49).

#### Within-Other Group Differences by Sex and Age

Different patterns of sex and age-related findings were noted in the other group. Males were more often diagnosed with schizophrenia/psychotic disorder and exceeded cut-offs related to cognition, aggression, mania, and positive symptoms, whereas this was true for anxiety and personality disorders, ADL impairment, and pain among females. In this group, the oldest more often had mood disorder diagnoses and exceeded cut-offs for aggression, depression, social withdrawal, and pain, while signs of mania, substance-related disorders, and personality disorders were most common in the youngest. A potential problem with substances was highest for those 25 to 54 years.

### Areas of Need

Table 4 reports on areas of need as measured by CAPs.

**Table 4:** Univariate distribution (%) for areas of need overall, and by WSIB status, sex, and age.

	**WSIB**						**Other**					
	**All**	**Sex**		**Age category**			**All**	Sex*		**Age category**		
		**F**	**M**	** <25**	**25-54**	**55+**		**F**	**M**	** <25**	**25-54**	**55+**
***N***	1,091	401	734	53	816	222	448,037	219,504	228,408	73,723	265,205	109,109
Harm to others^a,c,d,e^	20.71	19.49	21.40	39.62	19.11	22.07	32.96	27.70	38.02	38.92	32.37	30.37
Suicidality/purposeful self-harm^b,c,d,e^	41.34	47.95	37.66	60.38	42.90	31.08	38.59	42.06	35.24	46.74	39.98	29.69
Self-care^a,c,d,e^	34.10	35.39	33.38	47.17	40.99	31.37	51.14	48.94	53.26	47.58	47.51	62.34
Social relationships^b,d,e^	55.00	59.49	52.50	62.27	55.76	56.45	54.01	54.75	53.31	61.27	54.39	48.20
Supports for discharge^a,d,e^	27.13	27.95	26.68	26.42	25.61	32.88	33.37	30.83	35.80	30.20	33.59	34.97
Interpersonal conflict^b,d,e^	36.75	41.03	34.38	49.03	37.26	31.98	39.36	38.18	40.51	43.93	38.91	47.39
Traumatic life events^a,b,c,d,e^	25.48	30.00	22.97	26.41	28.18	15.31	15.58	19.94	11.37	16.12	17.06	11.60
Criminal activity^a,b,c,d,e^	22.18	14.10	26.68	30.19	23.65	14.86	32.15	22.35	41.57	37.52	35.16	21.21
Personal finances^a,c,d,e^	18.79	20.52	17.83	54.72	15.31	30.18	25.59	24.62	26.53	20.79	21.97	37.64
Education and employment^a,c,d,e^	30.89	31.28	30.67	33.96	33.83	19.37	33.90	30.75	36.91	57.98	35.86	12.85
Control interventions^a,d,e^	12.10	14.10	10.98	18.86	10.78	15.32	20.98	18.88	22.99	25.66	20.48	19.03
Medication management and adherence^a,c,d,e^	32.45	34.61	31.24	45.28	30.15	37.84	45.62	43.57	47.59	43.23	42.86	53.94
Rehospitalization^a,c,d,e^	29.61	29.23	29.81	49.06	30.52	21.62	42.18	41.25	43.07	39.31	44.47	38.56
Smoking^a,b,c,d,e^	50.23	43.84	53.78	49.06	54.41	35.13	52.37	36.22	49.13	43.45	49.32	26.54
Substance use^a,b,c,d,e^	58.02	47.95	63.62	75.47	61.64	40.54	46.92	39.28	54.27	63.76	50.96	25.73
Weight management^a,d,e^	46.57	50.25	44.50	43.40	47.91	42.34	37.45	43.87	31.29	33.54	38.66	37.18
Exercise^d,e^	24.29	25.39	23.68	20.75	23.40	28.38	23.80	24.99	22.67	19.69	21.61	31.90
Sleep disturbance^a,c,d,e^	48.95	51.03	47.79	33.96	50.74	45.94	37.12	39.11	35.19	33.64	37.37	38.84
Pain^a,c,d,e^	38.32	36.41	39.37	3.77	41.66	34.24	12.15	13.89	10.47	8.68	12.48	16.32
Falls^d,e^	8.74	8.02	9.11	0	8.19	3.51	5.47	6.11	4.87	4.48	10.56	2.64

* *An additional N = 125 identified their sex as “other”*;

a *Significant difference between the WSIB and Other group (p < 0.001)*;

b *Significant difference by sex within the WSIB group (p < 0.001)*;

c *Significant difference by age category within the WSIB group (p < 0.001)*;

d *Significant difference by sex within the Other group (p < 0.001)*;

e *Significant difference by age category within the Other group (p < 0.001)*.

#### Between Group Differences

CAPs for traumatic life events, substance use, weight management, sleep disturbance, and pain were more often triggered in the WSIB group; harm to others, self-care, supports for discharge, criminal activity, personal finances, education and unemployment, control interventions, medication management and adherence, rehospitalization, and smoking were more common in the comparison group. There were no differences for suicidality and purposeful self-harm (*p* = 0.06), social relationships (*p* = 0.52), interpersonal conflict (*p* = 0.07), exercise (*p* = 0.71), and falls (*p* = 0.09).

#### Within-WSIB Group Differences by Sex and Age

Females in the WSIB group more often had needs related to suicidality and purposeful self-harm, social relationships, interpersonal conflict, and traumatic life events. Males more often triggered CAPs for criminal activity, smoking, and substance use. There were no differences by sex for all other CAPs (*p*-values ranged from 0.07 to 0.84). The youngest group more frequently triggered almost all CAPs, including harm to others, suicidality and purposeful self-harm, self-care, criminal activity, personal finances, education and employment, medication management and adherence, rehospitalization, and substance use. Traumatic life events, smoking, sleep disturbance, and pain were most common among those aged 25 to 54 years. There were no age differences for social relationships (*p* = 0.20), support for discharge (*p* = 0.09), interpersonal conflict (*p* = 0.06), control interventions (*p* = 0.06), weight management (*p* = 0.30), exercise (*p* = 0.26), and falls (*p* = 0.20).

#### Within-Other Group Differences by Sex and Age

In the other group, the triggering of all CAPs differed by both sex and age. Different than what was found in the WSIB group, females in this group more frequently triggered CAPs for weight management, exercise, sleep disturbance, pain, and falls. Males more often had needs related to harm to others, self-care, supports for discharge, interpersonal conflict, personal finances, education and employment, control interventions, medication, and rehospitalization. The youngest group had additional needs around social relationships and control interventions, while this was true for rehospitalization, weight management, and falls among those 25-54 years. The oldest more often had needs related to self-care, supports for discharge, interpersonal conflict, personal finances, medication management and adherence, exercise, sleep disturbance, and pain.

## Discussion

This study reports on over a thousand Ontarians whose psychiatric admission was the responsibility—partially or fully—of the WSIB between 2006 and 2016; which is up to 12 years prior to its coverage for chronic mental stress. As such, it is suspected that WSIB was the payor due to a comorbid workplace physical injury or illness, or to severe traumatic experience. Approximately one quarter of individuals in this group had experienced trauma, and presumably, the remainder received coverage related to physical injuries. While those with WSIB coverage represent a small minority of psychiatric admissions, it has been a growing one. As the WSIB expanded coverage to chronic mental stress in 2018, it is expected that the number of admissions will continue to grow. Future studies should examine the profiles of persons admitted since the change in legislation.

In this study, the WSIB group tended to be comprised of males, which is consistent with the distribution of WSIB claims; for example, between 2006 and 2016, males represented 57–64% of all WSIB claims (21). Similar distributions have been reported in other provinces (22). The age distribution across all WSIB claims within the same time frame, however, is different. While the majority of claims were made by individuals between 40 and 54 years of age in both populations, those in this age bracket represented 40.35% of all WSIB claims and 49.86% of the study population. The biggest difference was seen amongst people under the age of 25, who represented 12.22% of claims and 4.51% of the study population (21). In Ontario, longer claim duration was associated with older age (23), and longer unemployment is associated with psychological distress (24). Future studies that use data linkage between WSIB claims and interRAI are needed to better understand the timing of injury, claims, and admissions. This would help to understand whether those in this study were older because they were older at the time of their injury, or because they were off of work for a longer duration and experienced negative mental health consequences because of longer unemployment.

The personal characteristics of the WSIB group were different than that of the comparison group. There were more people in the WSIB group who were married and living with a spouse than in the group not covered by the WSIB. This may help to explain the finding that fewer people in the WSIB group triggered for potential problems with availability of a support system after discharge, though it is important to note that more than one quarter did. Further, eligibility for coverage by WSIB means that these individuals had been employed, making them less likely to trigger the two CAPs related to economic issues. Again though, it needs to be noted that almost a third of individuals in the WSIB group did have issues related to education and employment, and so return to work—to either previous or new employment, or possibly retraining/education remain areas of concern.

In terms of the clinical characteristics and areas of need, the findings reported in this study are in line with the literature showing increased depression (24–26), substance use (27–29), and pain (30) among those who have experienced workplace injury. The literature has also described the co-occurrence and relationships between workplace injury, pain, depression, and substance use (30). While beyond the scope of this descriptive study, such relationships could easily be examined in the data. For example, subsequent analyses (not shown) revealed that 31.74% in the WSIB group experienced daily pain, and this increased to 47.88% among those admitted for addiction or dependency; that about two thirds of males in the WSIB group had needs related to substance use, and this increased to 72.46% among those with daily pain. That pain, depression, and substance use each represent chronic conditions provide impetus for further exploration of these relationships in the data cross-sectionally and longitudinally. Given that the interRAI instrument also collects information related to service use and is completed at the time of discharge, evaluation of adequate pain management on outcomes and the extent to which it may potentially reduce the likelihood or severity of mental health issues are possible, as is looking at impact on length of stay.

The use of sex- and age-based analyses further describes the specific needs of injured workers admitted to inpatient psychiatry. In particular, that almost three quarters of females in the WSIB group showed signs of clinically relevant depression, as well as more traumatic life events and issues related to social relationships, interpersonal conflict, and suicidality and purposeful self-harm represents a very different profile than males. Many of these needs were also shown to be more prevalent in the youngest group (i.e., under 25 years). More specifically, they had more needs related to safety (suicidality and purposeful self-harm, harm to others, self-care), social life (criminal activity), economic issues (personal finance, education and employment), and autonomy (medication management and adherence, rehospitalization), and displayed more severe aggression, positive symptoms, and issues with substances. While in and of itself, it is not surprising that the youngest group would more often experience some of these issues (e.g., related to education, personal finances), it is somewhat surprising that they are experiencing higher rates of *most* issues assessed in the instrument. While a recent systematic review examined associations between work-related stressors and the mental health of young workers (31), to our knowledge, the focus of research on younger injured workers remains largely on the physical aspects of the injuries. This study points to the need for additional focus on the mental health needs and outcomes among young injured workers. Further research that examines clinical needs among males and females of different ages is recommended. In addition, longitudinal research is needed on outcomes over time, including rehospitalization, to better understand the factors influencing recovery. That just under half of those under the age of 25 years were at risk for rehospitalization further points to the complexity of issues facing this group and the need to intervene as soon as possible.

The interRAI instrument contains over 300 items on the strengths, preferences, needs, and service use in inpatient psychiatry, but it does not cover work-related disability specifically. As such, it does not provide information on the timing or nature of the work-related injury. Similarly, the instrument assesses for presence of traumatic events, depression, self-harm, substance us, and pain, for example, but does not have specific information on the timing of onset. Intersectoral collaboration is needed to develop mechanisms to link WSIB and inpatient psychiatry data to better understand the mental health needs of injured workers, and to explore the causal pathways involved.

A major strength of this study is the scope of variables included. The instrument's items, scales, and CAPs have demonstrated reliability and validity, and allow researchers to analyze a range of characteristics, thus providing a comprehensive description of the study population. A second strength is the population-based nature of the data. All persons admitted into a psychiatric facility or psychiatric unit in a hospital are required to be assessed with this instrument. Therefore, the entire population of interest is included in this study. A limitation, however, is that there is no way to know why the person was covered by the WSIB, nor when this occurred. They could have had a physical injury or a severe traumatic experience at work that resulted in work disability, which could have happened 5 years ago or 1 year ago. As stated previously, future studies linking WSIB claims and interRAI inpatient psychiatry population data are recommended. This will become increasingly important given the 2018 policy change to include coverage for chronic mental stress. Another drawback includes the inability to assess the timeline of the mental illness that led to the admission of the individual at a psychiatric facility and the injury that resulted in WSIB coverage. It would be interesting to see the effects of time off work on mental health illness, as well as the order of events between work injury and mental health illness.

## Conclusion

This paper provides a description of individuals who had their inpatient psychiatric stay paid for by WSIB (at least partially) over an 11-year period. Future studies should examine impacts of the policy introduced in January 2018 to determine whether changes are observed in the characteristics and needs of individuals admitted to inpatient psychiatry for (or related to) work-related mental health injuries. Sex- and age-based analyses are also needed to further elucidate the relationships observed. Data linkage with WSIB claims would allow further understanding of the circumstances of the workplace injury, as well as potentially enable understanding of causal pathways between workplace injury and mental health.

## Data Availability Statement

The data analyzed in this study is subject to the following licenses/restrictions: The data are made available to interRAI Fellows for research use under an existing license agreement interRAI has with the Canadian Institute for Health Information; note that the agreement is for research only, not commercial use. Students working under the supervision of an interRAI Fellow can apply for free access to the data, but are subject to terms of use. As part of interRAI's agreement with the Canadian Institute for Health Information, the data may not be transmitted to third parties; therefore, the data used in this study cannot be made available to others. Those interested in using the data can apply directly to the Canadian Institute for Health Information for access. Requests to access these datasets should be directed to https://www.cihi.ca/en/access-data-and-reports/make-a-data-request.

## Ethics Statement

Ethical review and approval was not required for the study on human participants in accordance with the local legislation and institutional requirements. The ethics committee waived the requirement of written informed consent for participation.

## Author Contributions

MH conducted the analyses of the data, with input from the LM and VK. MH drafted the initial version of the manuscript, with major writing contributions by the LM. VL finalized the manuscript. All authors contributed to the development of the study topic and design, and provided valuable and important feedback on the manuscript to its completion.

## Conflict of Interest

The authors declare that the research was conducted in the absence of any commercial or financial relationships that could be construed as a potential conflict of interest.
